# Regulation of gene expression by the APP family in the adult cerebral cortex

**DOI:** 10.1038/s41598-021-04027-8

**Published:** 2022-01-07

**Authors:** Hye Ji Cha, Jie Shen, Jongkyun Kang

**Affiliations:** 1 Division of Hematology/Oncology, Boston Children’s Hospital and Department of Pediatric Oncology, Dana-Farber Cancer Institute (DFCI), Harvard Stem Cell Institute, Harvard Medical School, Boston, MA 02115 USA; 2 Department of Neurology, Brigham and Women’s Hospital, Harvard Medical School, Boston, MA 02115 USA; 3 Program in Neuroscience, Harvard Medical School, Boston, MA 02115 USA

**Keywords:** Neuroscience, Diseases of the nervous system, Molecular neuroscience, Transcriptomics

## Abstract

Amyloid precursor protein (APP) is associated with both familial and sporadic forms of Alzheimer’s disease. APP has two homologs, amyloid precursor-like protein 1 and 2 (APLP1 and APLP2), and they have functional redundancy. APP intracellular c-terminal domain (AICD), produced by sequential α- or β- and γ-secretase cleavages, is thought to control gene expression, similarly as the ICD of Notch. To investigate the role of APP family in transcriptional regulation, we examined gene expression changes in the cerebral cortex of *APP/APLP1/APLP2* conditional triple knockout (cTKO) mice, in which APP family members are selectively inactivated in excitatory neurons of the postnatal forebrain. Of the 12 previously reported AICD target genes, only *Nep* and *Npas4* mRNA levels were significantly reduced in the cerebral cortex of cTKO mice, compared to littermate controls. We further examined global transcriptional changes by RNA-seq and identified 189 and 274 differentially expressed genes in the neocortex and hippocampus, respectively, of cTKO mice relative to controls. Gene Ontology analysis indicated that these genes are involved in a variety of cellular functions, including extracellular organization, learning and memory, and ion channels. Thus, inactivation of APP family alters transcriptional profiles of the cerebral cortex and affects wide-ranging molecular pathways.

## Introduction

Alzheimer’s disease (AD) is the most common cause of dementia and the sixth leading cause of death in the United States. Amyloid precursor protein (APP) was the first protein identified to be associated with AD, and since the molecular cloning of full-length APP cDNA, its physiological function has been extensively studied^1–3^. After the first mutation was discovered in the transmembrane domain of APP in familial AD (FAD)^4^, several additional pathogenic FAD mutations around the α-, β- or γ-secretase cleavage sites of APP have been identified (alzforum.org/mutations/app), highlighting the importance of the endoproteolytic processing of APP in AD pathogenesis.

APP is a type I transmembrane protein that is proteolytically processed into multiple different fragments. The initial cleavage of APP by either α-secretase (non-amyloidogenic pathway) or β-secretase (amyloidogenic pathway) within the Aβ region generates the N-terminal soluble ectodomains APPsα and APPsβ and the membrane-integrated C-terminal fragments αCTF or βCTF, respectively. The CTF fragments are subsequently cleaved by γ-secretase and produce Aβ, p3, and APP intracellular c-terminal domain (AICD)^5^. In mammals, *APP* has two homologs, amyloid precursor-like proteins 1 and 2 (*APLP1* and *APLP2*)^6,7^, which are similarly processed by the same secretases, producing the intracellular fragments of APLPs, termed ALID (APLP-intracellular domain) 1 and ALID2^8^.

Notably, APP processing is highly reminiscent of Notch processing, which triggers translocation of the Notch intracellular domain to the nucleus by γ-secretase and results in the transcriptional regulation of defined target genes involved in neuronal differentiation^9–11^. Thus, a similar functional role has been proposed for AICD, ALID1 and ALID2 as transcriptional regulators^8,12,13^. Indeed, AICD has been shown to translocate to the nucleus and can form a complex with the adaptor FE65 and the histone acetyltransferase Tip60 in cultured cells^12,14^. In addition, other putative AICD or ALIDs downstream target genes, including *Kai1*^15^, *GSk3b*^16^, *Bace1*, *Tip60*^17^, *Nep*^18^, *p53*^19^, *Egfr*^20^, *Lrp*^21^, and *Npas4*^22^, have been reported in APP germline knockout mice or in vitro culture systems. However, the validity of these proposed targets, and particularly whether they are also endogenous downstream targets of AICD or ALIDs, has remained controversial due to the lack of an adequate animal model.

To provide molecular insight into the physiological and signaling function of APP family members in the adult brain, we used recently generated *APP/APLP1/APLP2* triple conditional knockout (cTKO) mice^23^, in which all APP family members are selectively inactivated in excitatory neurons postnatally in the forebrain. We evaluated the expression of previously reported putative downstream target genes of AICD or ALIDs in the neocortex and hippocampus at the age of 3 months. In addition, unbiased analysis of global gene expression using RNA-seq revealed that loss of the APP family alters the transcription profile in the adult mouse cerebral cortex. These findings represent an initial step toward a more complete understanding of the role of APP family genes in the adult brain.

## Results

### Downregulation of the expression levels of *Nep* and *Npas4* in the cerebral cortex of cTKO mice

**Figure 1:**
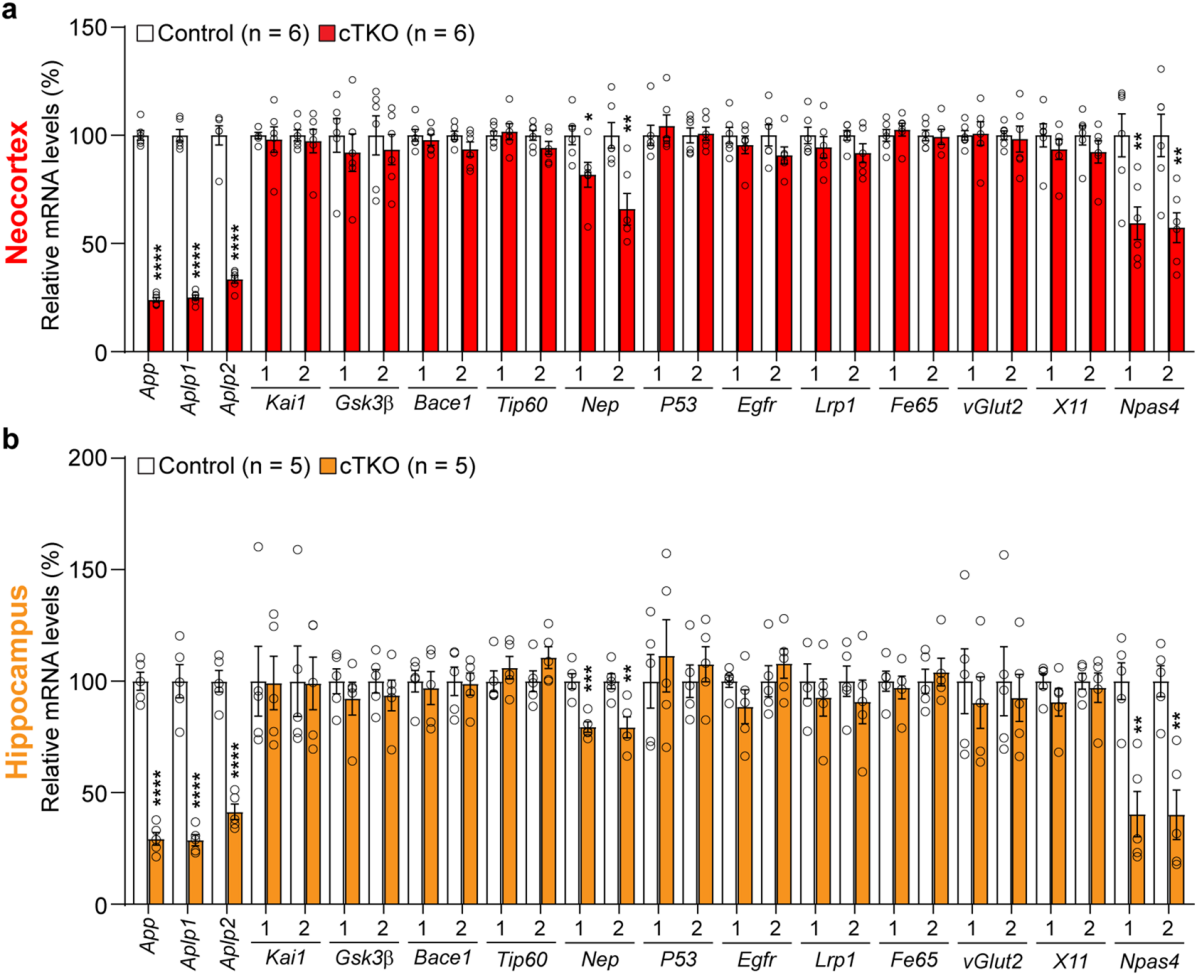
Expression levels of *APP* family genes and putative AICD target genes in the cerebral cortex of cTKO mice at 3 months of age. (**a**,**b**) Relative mRNA levels of putative AICD target genes in the neocortex (**a**) and hippocampus (**b**) of cTKO mice relative to the controls at 3 months of age. The mRNA levels of each gene were normalized to the mRNA levels of the *Gapdh* and *Rpl13a* housekeeping genes. All data are expressed as the means ± SEMs. n = 5–6 mice per genotype. Statistical analysis was performed with Student’s *t*-test. *p < 0.05, **p < 0.01, ***p < 0.001, ****p < 0.0001.

Our first goal was to determine whether the expression levels of previously identified putative AICD downstream target genes are changed in the absence of APP, APLP1, and APLP2 in the adult brain. Due to the genetic redundancy of APP, APLP1 and APLP2 and the perinatal lethality of *APP/APLP1/APLP2* germline triple knockout (TKO) mice, it is not possible to study the function of the APP family in the adult brain^24^. Thus, we used *APP/APLP1/APLP2* conditional TKO (*fAPP*/*fAPP; fAPLP1/fAPLP2; fAPLP2/fAPLP2; Camk2a-Cre*; hereafter cTKO) mice, in which all APP family members are selectively inactivated in excitatory neurons postnatally in the forebrain^23^. These cTKO mice show mild learning and memory deficits as well as impairment of synaptic plasticity at the age of 3 months, but there is no gross abnormality in the cerebral cortex of cTKO up to ~ 2 years of age. We performed qRT-PCR of total RNA extracts from the neocortex and hippocampus of cTKO and control mice (*fAPP*/*fAPP; fAPLP1/fAPLP2; fAPLP2/fAPLP2*) at age 3 months. Consistent with previous Northern analysis results^23^, we observed a significant decrease in the mRNA expression of *App*, *Aplp1*, and *Aplp2* in the neocortex (*App*: 76%, p < 0.0001; *Aplp1*: 73%, p < 0.0001, *Aplp2*: 67%, p < 0.0001; Fig. 1a) and hippocampus (*App*: 71%, p < 0.0001; *Aplp1*: 71%, p < 0.0001; *Aplp2*: 59%, p < 0.0001, Student’s *t*-test, n = 5 per genotype; Fig. 1b) of cTKO compared to littermate control mice.

Then, we evaluated the expression levels of previously reported putative AICD downstream target genes (*Kai, Gsk3b, Bace1, Tip60, Nep, P53, Egfr, Lrp1, Fe65, vGlut2, X11,* and *Npas4*)^12,14–22^ in the cerebral cortex of cTKO and control mice by qRT-PCR analysis. We detected a significant decrease in the levels of *Nep* (1st primer set: 18%, p = 0.0296; 2nd primer set: 34%, p = 0.0046, Student’s *t*-test) and *Npas4* (1st primer set: 41%, p = 0.0086; 2nd primer set: 43%, p = 0.0053, Student’s *t*-test) in the neocortical transcriptome in cTKO mice compared to controls (Fig. 1a). Similarly, the expression levels of *Nep* and *Npas4* in the hippocampus were significantly decreased in cTKO mice compared to controls (*Nep*; 1st primer set: 21%, p = 0.0009; 2nd primer set: 21%, p = 0.0076, *Npas4*; 1st primer set: 60%, p = 0.0019; 2nd primer set: 60%, p = 0.0018, Student’s *t*-test, Fig. 1b). However, the expression levels of other putative AICD downstream target genes showed no significant difference between cTKO and control mice. Together, loss of all APP family genes in the cerebral cortex in vivo reduces gene expression of *Nep* and *Npas4*, which have been reported as AICD target genes in other animal model systems.

### Differential gene expression in the cerebral cortex of mice lacking APP family gene in excitatory neurons

**Figure 2:**
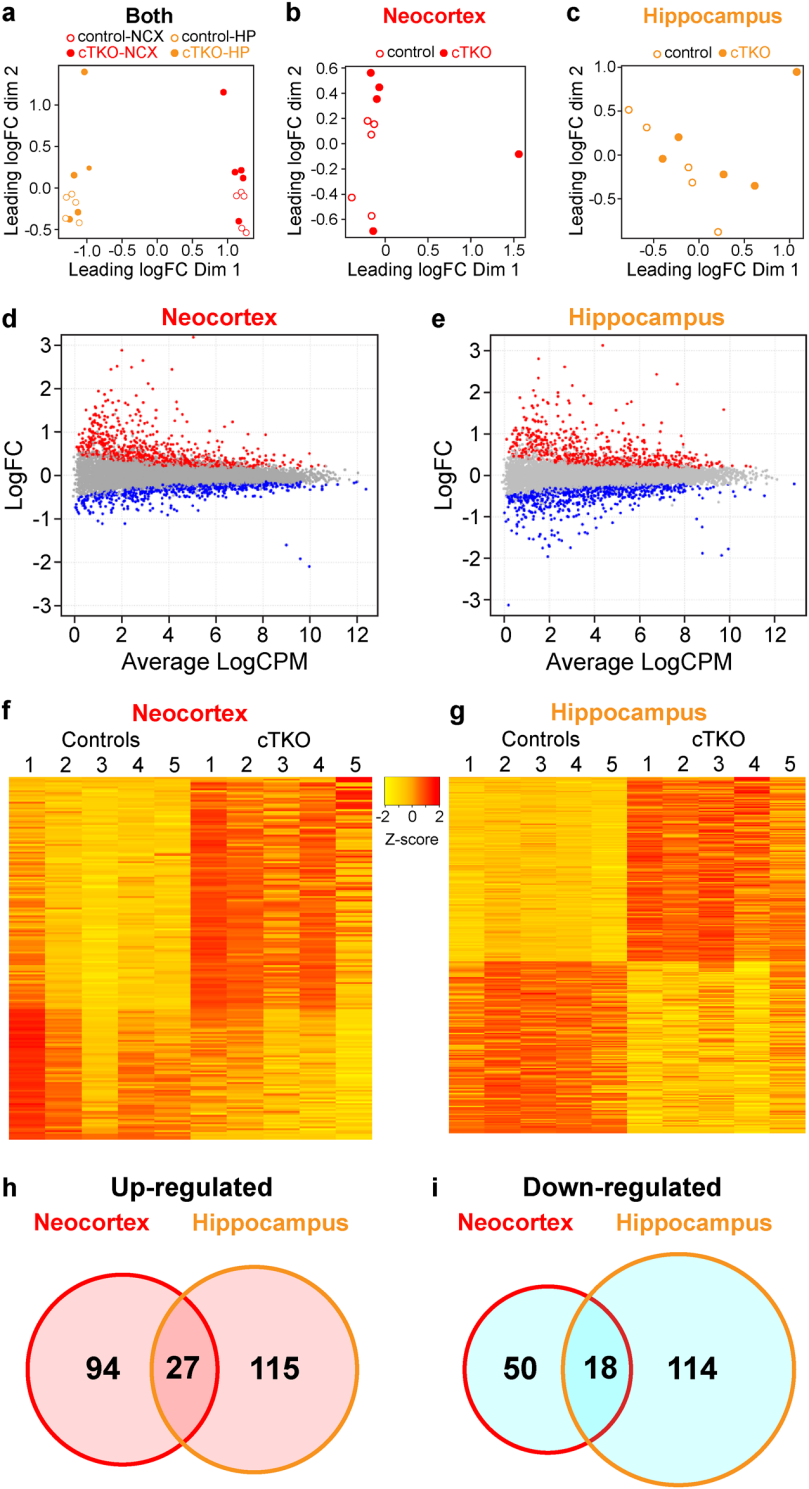
Significant changes in the transcriptome profile of the cerebral cortex by loss of APP family in the excitatory neurons. (**a**–**c**) Multi-dimensional scaling (MDS) plots of the RNA-seq data from all 20 samples (**a**), the neocortex (**b**), and hippocampus samples (**c**). n = 5 mice per genotype. (**d**,**e**) MA plots showing the (log2) normalized mean of all genes expressed across the control samples plotted against the neocortex (**d**) or hippocampus (**e**) of cTKO mice at the age of 3 months. The upregulated genes (p-value < 0.05) are shown in red, and the downregulated genes (p-value < 0.05) are shown in blue. (**f**,**g**) The relative RNA expression levels of all significant DEGs (FDR p-value < 0.05) in the neocortex (**f**) or hippocampus (**g**) between controls and cTKO mice were calculated as z-scores and displayed in heatmaps. The color scale of the z-scores’ (− 2, yellow, to + 2, red) is shown between the panels. (**h**,**i**) Venn diagrams show the overlaps among the up- or down-regulated DEGs in the neocortex (**h**) and hippocampus (**i**) between the controls and cTKO mice.

Having confirmed that loss of APP family genes can alter gene expression of *Nep* and *Npas4* in the cerebral cortex, we then aimed to assess the global impact of APP family gene loss on transcriptional changes by performing RNA-seq experiments using the neocortex and hippocampus of cTKO and littermate control mice (n = 5 per genotype) at the age of 3 months. A total of 86–91% of raw reads were mapped to the mm10 mouse genome (Supplementary Table 1). Multi-dimensional scaling (MDS) plots indicated that gene expression profiles were more distinct between different tissues than genetic changes of between cTKO mice and controls (Fig. 2a–c).

We then compared the gene expression in the neocortex and hippocampus of cTKO mice and littermate controls using edgeR^25^. Comparison of the neocortex transcriptomes of cTKO mice and littermate controls identified 189 differentially expressed genes (DEGs) (121 up-regulated and 68 down-regulated in cTKO) with a Fales Discovery Rate (FDR) of less than 5% (Fig. 2d,f). In the hippocampus, a total of 274 DEGs (132 up-regulated and 142 down-regulated in cTKO) were identified with an FDR of less than 5% (Fig. 2e,g). Consistent with our qRT-PCR data, the expression levels of several putative AICD target genes (*Kai, Gsk3b, Bace1, Tip60, P53, Egfr, Lrp1, Fe65, vGlut2,* and *X11*) were not changed in either hippocampal or neocortical transcriptome of cTKO mice compared to those of the controls. In contrast, the expression level of *Npas4* was significantly decreased in the neocortex (LogFC = − 0.81, FDR p-value < 0.0001) and hippocampus (LogFC = − 1.38, FDR p-value < 0.0001) of cTKO mice. For *Nep*, while the change in its expression level in cTKO mice compared to the controls was not significant for the whole group (NCX: LogFC = − 0.06, FDR p-value = 0.97; HP: LogFC = − 0.38, FDR p-value = 0.54), significantly reduced expression of *Nep* in the hippocampus was observed when the data from one cTKO mouse with the most different gene expression profile were removed (LogFC = − 0.65, FDR p-value = 0.007).

**Figure 3:**
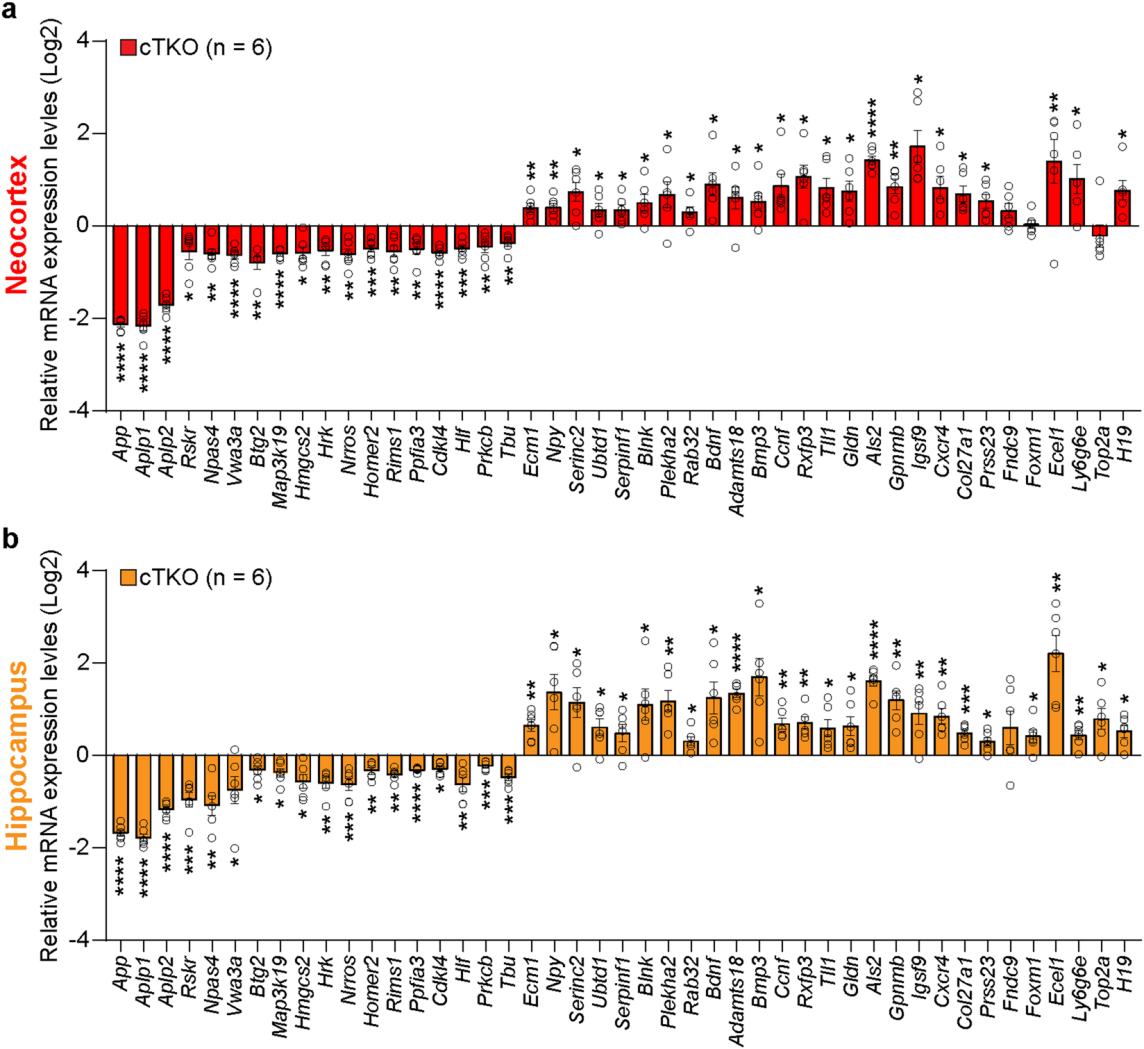
Validation of common DEGs in the neocortex and hippocampus. (**a**,**b**) Quantification of relative gene expression levels of the RNA-seq common DEGs in the neocortex (**a**) and hippocampus (**b**) of cTKO mice compared to controls at the age of 3 months. The mRNA levels of each gene were normalized to the mRNA levels of the *Gapdh* and *Rpl13a* housekeeping genes. All data are expressed as the means ± SEMs. Statistical analysis was performed with Student’s *t*-test. *p < 0.05, **p < 0.01, ***p < 0.001, ****p < 0.0001.

Further examination of DEGs revealed that 27 genes were upregulated (*Adamts18, Als2, Bdnf, Blnk, Bmp3, Ccnf, Col27a1, Cxcr4, Ecel1, Ecm1, Fndc9, Foxm1, Gldn, Gpnmb, H19, Igsf9, Ly6g6e, Npy, Plekha, Prss23, Rab32, Rxfp3, Serinc2, Serpinf1, Tll1, Top2a,* and *Ubtd1*) and 18 genes were downregulated (*App, Aplp1, Aplp2, Rskr, Btg2, Cdk14, Hlf, Hmgcs2, Homer2, Hrk, Map3k19, Npas4, Lrrc33, Ppfia3, Prkcb, Rims1, Tub,* and *Vwa3a*) in both the neocortex and hippocampus of cTKO mice compared to the controls (Fig. 2h,i, Supplementary Table S2). We then performed technical validation of our RNA-seq dataset by qRT-PCR of cerebral cortex obtained from a different set of mice. First, we confirmed that *App*, *Aplp1*, and *Aplp2* expression was, as expected, significantly lower in cTKO mice than in the controls (Fig. 3a,b). We performed qRT-PCR for the set of DEGs that were differentially expressed in both the neocortex and hippocampus (42 genes). The qRT-PCR results showed that of these 42 genes, 39 and 41 showed identical patterns to those determined by RNA-seq in the neocortex and hippocampus, respectively. However, for 3 genes (*Fndc9, Foxm1, Top2a*), although qRT-PCR confirmed the direction of the changes in mean expression (higher or lower) in cTKO mice, the difference was not statistically significant. Thus, our validation rate was 93% for the neocortex and 98% for the hippocampus. This finding suggests that the expressions of these genes are affected by APP family signaling universally with spatial restriction. Overall, these data demonstrate that the inactivation of the APP family in the cerebral cortex alters the transcription profile of a large set of genes with diverse roles.

### Changes of molecular pathways in the cerebral cortex by the loss of APP family

**Figure 4:**
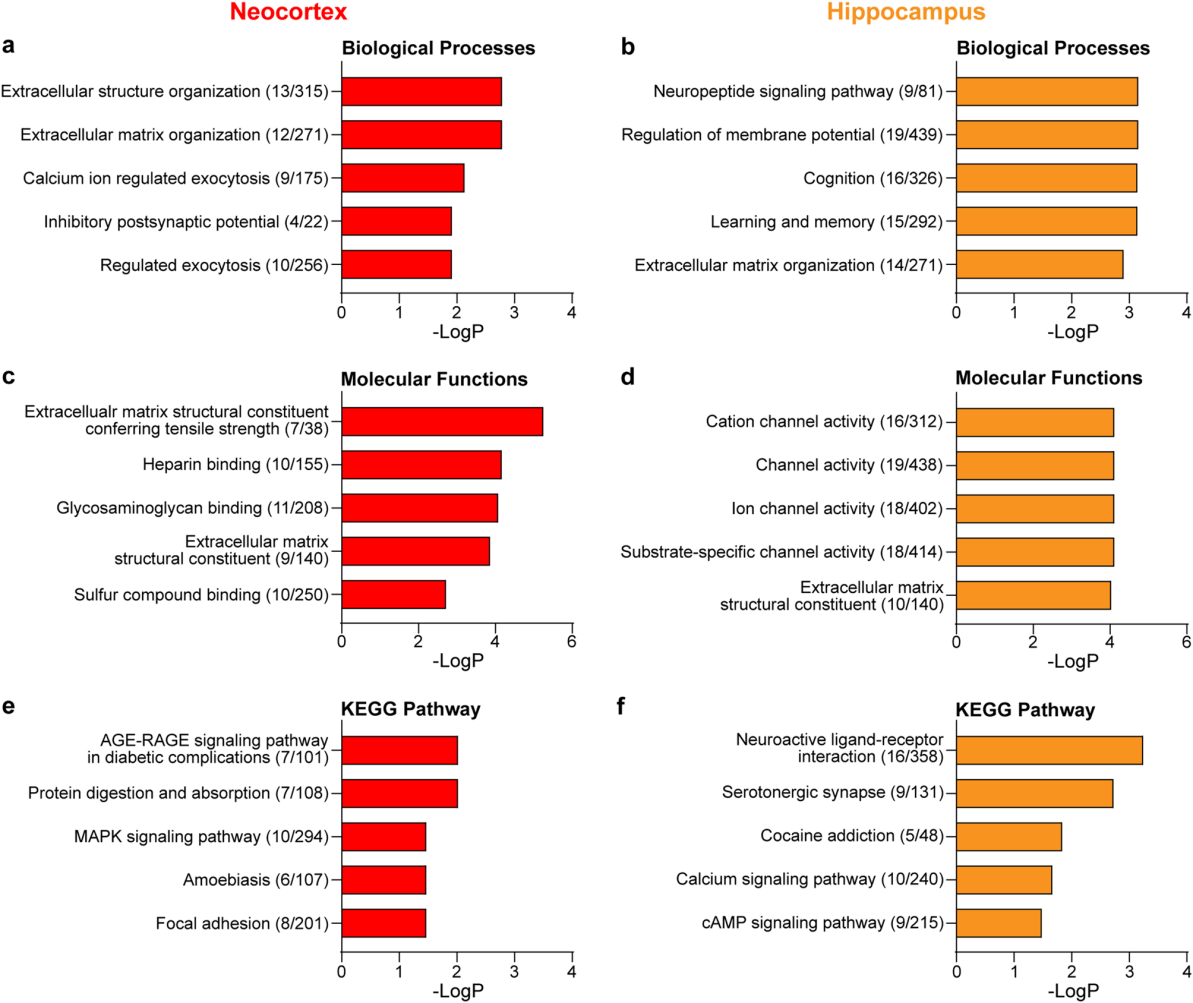
GOs enriched for DEGs in the cerebral cortex between controls and cTKO mice. (**a**,**b**) The top 5 significantly enriched Biological Processes GO terms in the neocortex (**a**) and hippocampus (**b**) of cTKO mice. (**c**,**d**) The top 5 significantly enriched Molecular Functions GO terms in the neocortex (**c**) and hippocampus (**d**) of cTKO mice. (**e**,**f**) The top 5 significantly enriched KEGG Pathways in the neocortex (**e**) and hippocampus (**f**) of cTKO mice. The bars depict the negative logarithm of the false discovery rate (FDR). The numbers in parentheses indicate the number of DEGs and the total number of genes in the cluster.

To explore the biological processes and molecular functions of the genes that were differentially regulated in the cerebral cortex of cTKO mice compared to that of controls, we performed Gene Ontology (GO) enrichment analyses using the Goseq R package^26^. The GO terms enriched among the DEGs with FDR-corrected p-values of less than 0.05 were identified. The DEGs in the neocortex and hippocampus were annotated with 86 and 133 GO biological process terms, respectively. In particular, the extracellular structure organization, calcium ion regulated exocytosis, and inhibitory postsynaptic potential were top enriched pathways in biological processes affected by the loss of APP family in the neocortex (Fig. 4a). Interestingly, genes associated with the neuropeptide signaling pathway, regulation of membrane potential, and learning- or memory-related biological processes were most enriched pathways in the hippocampus (Fig. 4b). In parallel, a total of 20 (neocortex) and 53 (hippocampus) GO molecular function terms were identified with an FDR-corrected p-value of less than 0.05. Consistent with the biological processes GO terms, extracellular matrix structural constituents and channel activity were observed as most changed in the neocortex and hippocampus, respectively (Fig. 4c,d).

We also performed Kyoto Encyclopedia of Genes and Genomes (KEGG) pathway analysis^27^ to investigate whether the DEGs associated with specific biochemical pathways. Among the 189 DEGs in the neocortex, significant enrichment was found in five KEGG pathways (adjusted p-value < 0.05). The most significantly overrepresented pathway was the AGE-RAGE signaling pathway in diabetic complications. Similarly, significant enrichment of protein digestion and absorption and the MAPK signaling pathway was observed (Fig. 4e). Among the hippocampus DEGs, nine KEGG pathways (adjusted p-value < 0.05) were significantly enriched. Interestingly, neuroactive ligand-receptor interactions and calcium signaling pathways were significantly overrepresented (Fig. 4f). Overall, our GO analysis indicates that loss of the APP family genes in excitatory neurons of the cerebral cortex has very significant impacts on the extracellular structure, ion channel activity, and neuropeptide signaling processes.

## Discussion

Transcriptome analysis of the postmortem human brain tissue^28–32^ or genetically modified animal models^33–38^ has provided a comprehensive overview of the transcriptional responses associated with AD pathogenesis. In particular, AICD has been proposed as a key epigenetic regulator of gene expression controlling a diverse range of genes. However, the validity of its proposed targets, and particularly regarding the question of whether they also constitute endogenous AICD targets in the adult brain, has remained controversial due to the presence of functional compensation of two other APP homologs, APLP1 and APLP2, and the lack of adequate animal models^39^. The creation of a new animal model, in which all APP family genes are selectively inactivated in excitatory neurons in the adult mouse brain, allowed us to investigate the changes in gene expression in the adult cerebral cortex, and the functions of APP family genes at the global level during their normal function. The qRT-PCR analysis revealed that among the proposed AICD interacting target genes, only *Nep* and *Npas4* exhibited significantly changed expression in the cerebral cortex of cTKO mice compared to controls. APP dependent expression changes of *Nep* or *Npas4* were previously investigated in the primary cortical neurons, fibroblasts or brains of APP knockout mice^18,22^, demonstrating that our findings are highly consistent with the results of previous studies using in vivo models. Conversely, the expression levels of other proposed target genes, which were previously identified and tested using in vitro culture systems, were similar in cTKO mice and controls.

Our RNA-seq analysis provides a comprehensive understating of the changes in transcriptional regulation that occur in the cerebral cortex in the absence of the APP family in excitatory neurons. Based on the 189 and 274 DEGs found in the neocortex and hippocampus, respectively, we performed an enrichment analysis using the KEGG and GO databases. The list of pathways obtained from this analysis may help elucidate the molecular mechanisms of phenotypes found in knockout models of the APP family. For example, we previously reported that the APP family regulates the synaptic plasticity and neuronal excitability through Kv7 channels in the hippocampal Schaffer Collateral synapses and CA1 neurons^23^. Consistent with these results, we found that membrane potential- and ion channel activity-related gene sets, particularly voltage-gated potassium channel-encoding genes (e.g. *Kcna5, Kcne1l, Kcnh5,* and *Kcnq3* etc.), were enriched in the hippocampus. Interestingly, the mRNA levels of *Kcnq3*, one of Kv7 encoding genes, were significantly decreased in the hippocampal transcriptome of cTKO mice compared to controls. The qRT-PCR analysis confirmed that only the expression levels of *Kcnq3*, but not other Kv7 channel encoding genes, were significantly reduced in the hippocampus of cTKO mice relative to controls (Supplementary Fig. S1). The molecular pathway through which the APP family regulates Kv7 channel transcription and activity during the regulation of neuronal excitability is worth further investigation, and its elucidation may be further explored to identify novel therapeutic targets in AD.

APP family genes are highly expressed in excitatory neurons of the cerebral cortex but are not exclusive to these cells, as their gene products persist at some level in excitatory neuron-specific cTKO mice^23^. APP is also expressed in other types of cells, such as GABAergic interneurons and glial cells^40,41^. In addition, various protein fragments derived from APP regulate synaptic plasticity and neural networks^42–46^. Thus, we cannot exclude that cell-nonautonomous effects of the loss of APP family function in excitatory neurons on other types of cells have been observed. For example, the expression levels of *Npy*, which is secreted from GABAergic interneurons^47^, were significantly increased in the hippocampal transcriptome of cTKO mice. Hence, it would be interesting to investigate the cell type specific transcriptional changes that occur in the absence of the APP family at higher resolution by single-cell RNA-seq or by generating and analyzing inhibitory neuron- or glia-specific cTKO mice.

In conclusion, excitatory neuron-specific deletion of the APP family in the adult brain leads to changes in gene expression involved in a wide range of molecular pathways. The presented model can be used in future studies to investigate the interactions of the APP family with voltage-gated potassium channels as well as influence of APP family genes on other types of cells. Our work provides the first description of the transcriptome in cTKO mice. We expect this will provide a starting point for better understanding the function of APP family genes in the cerebral cortex.

## Methods

### Animals

All animal use was approved by the IACUC committee of Harvard Medical School and Brigham and Women's Hospital in accordance with the USDA Animal Welfare Act and PHS Policy on Humane Care and Use of Laboratory Animals. The study was carried out in compliance with the ARRIVE guidelines (http://arriveguidelines.org). The mice were maintained under a 12-h light/dark cycle, and were provided with standard rodent chow and water ad libitum. Mice of both sexes at 3 months of age were used for analysis. Floxed *APP/APLP1/APLP2* (*fAPP*/*fAPP; fAPLP1/fAPLP2; fAPLP2/fAPLP2*) mice were previously described^23^, and *Camk2a-Cre* transgenic mice were previously described^48^. The *APP/APLP1/APLP2* cTKO (*fAPP*/*fAPP; fAPLP1/fAPLP2; fAPLP2/fAPLP2; Camk2a-Cre*) and littermate control mice (*fAPP*/*fAPP; fAPLP1/fAPLP2; fAPLP2/fAPLP2*) were maintained on the C57BL/6 J and 129 hybrid genetic background and were obtained by breeding *fAPP*/*fAPP; fAPLP1*/*fAPLP1*; *fAPLP2*/*fAPLP2; Camk2a-Cre* or *fAPP*/+; *fAPLP1*/*fAPLP1*; *fAPLP2*/*fAPLP2; Camk2a-Cre* mice with *fAPP*/*fAPP; fAPLP1*/*fAPLP1*; *fAPLP2*/*fAPLP2* mice.

### Isolation of the neocortex and hippocampus

Mice were euthanized using the controlled release of carbon dioxide followed by cervical dislocation, and the brains were removed. The neocortex and hippocampus were dissected separately from the sagittal cut brain on ice and stored at − 80 °C until further processing.

### Total RNA extraction and qRT-PCR

Total RNA was extracted from the neocortex and hippocampus using the RNeasy Plus Universal Kit and RNeasy Plus Mini kit (Qiagen), respectively, according to the manufacturer’s instructions. Approximately 1 µg of RNA was reverse transcribed using iScript Reverse Transcription Supermix (Bio-Rad) according to the manufacturer’s instructions. For qPCR, each 10 µl reaction contained 5 µl of PowerUP SYBR Green PCR Master Mix (Thermo Fisher Scientific), 100 nM of each primer, and template cDNA corresponding to 250 ng of RNA. cDNA was denatured at 95 °C for 2 min and amplified for 45 cycles with a QuantStudio 6 (Thermo Fisher Scientific) using a standard protocol. The sequences of the primers can be found in Supplementary Table S3. The specificity of the primers was determined by the single peak in the melt curve. All reactions were performed in at least duplicates. The gene expression levels were normalized to the expression levels of *Gapdh* and *Rpl13a*.

### RNA-seq analysis

The neocortex and hippocampus were collected separately from five cTKO and five littermate controls at the age of 3 months, and total RNA was extracted as described above. The RNA quality of 20 samples was analyzed with a 2100 BioAnalyzer (Agilent Technologies) using the RNA 6000 Nano kit (Agilent Technologies), and all 20 samples had an RNA integrity number (RIN) between 8.1 and 9.8 (Supplementary Table S1)^49^. To enrich mRNAs from the total RNA sample (200 ng–1 µg per sample), poly-A pull-down was performed, and library preparation was conducted using an Illumina TruSeq RNA prep kit (Illumina). The libraries were then sequenced using Illumina HiSeq2500/HiSeq4000 with 100-bp single-end reads per sample at Columbia Genome Center (https://systemsbiology.columbia.edu/genome-center). The entire RNA-seq dataset is available at the Gene Expression Omnibus (GEO) database with the accession number GSE181936.

The sequencing reads were mapped to the mm10 mouse reference genome using STAR (v2.5.4a)^50^ with the default parameters. Raw counts of the aligned reads were produced using HTseq^51^. Differential expression analyses were then performed as described^52^ using edgeR^25^ to identify significantly differentially expressed genes in the neocortex and hippocampus tissues between cTKO mice and controls. After the trimmed mean of M-values (TMM) normalization, multi-dimensional scaling (MDS) plots were generated based on leading log-fold-change distances and the first two dimensions were selected to illustrate the data.

### Enrichment analysis of gene ontology (GO) terms

DEGs (FDR < 0.05) in cTKO mice compared to the control mice were selected for enrichment analyses of gene sets. The Goseq R package^26^ was used to determine the enrichment of GO terms and KEGG pathways taking into account gene length bias. Adjusted p-values were calculated using the Benjamini–Hochberg method, and GO gene sets with p-values less than 0.05 were considered significant. In the KEGG pathway enrichment analysis, gene sets with p-values less than 0.05 were considered significant.

## Supplementary Information

Supplementary Information.

## Data Availability

The entire RNA-seq dataset is available at the Gene Expression Omnibus (GEO) database with the accession number GSE181936. Other datasets generated during and/or analyzed during the current study are available from the corresponding author on reasonable request.
