# Supplementary material for: Regulation of gene expression by the APP family in the adult cerebral cortex
Source: Sci Rep. 2022 Jan 7;12:66. doi: 10.1038/s41598-021-04027-8 (PMC8741778; doi:10.1038/s41598-021-04027-8)
Supplement: Supplementary file 1 — Supplementary Information. [file 41598_2021_4027_MOESM1_ESM.pdf]

Supplementary Information

Regulation of gene expression by the APP family in the adult cerebral cortex

Hye Ji Cha, Jie Shen, and Jongkyun Kang

1) Supplementary Table S1
2) Supplementary Table S2
3) Supplementary Figure S1
4) Supplementary Table S3

	RIN	Number of input reads	Number of uniquely mapped reads	Percentage of uniquely mapped reads
**Neocortex**				
Control 1	9.6	34886565	31744914	90.99%
Control 2	9.8	24389920	21925932	89.90%
Control 3	8.1	17582967	15857094	90.18%
Control 4	8.6	22801453	20413272	89.53%
Control 5	9.1	21320896	19162851	89.88%
cTKO 1	8.5	31272184	28441499	90.95%
cTKO 2	8.7	29265688	26340178	90.00%
cTKO 3	8.5	23139692	20616224	89.09%
cTKO 4	9.0	27636580	24831316	89.85%
cTKO 5	8.9	19242730	17120400	88.97%
**Hippocampus**				
Control 1	9.4	27674630	24394426	88.15%
Control 2	9.6	33684563	29293392	86.96%
Control 3	8.7	33162854	28511942	85.98%
Control 4	9.6	30798851	26951482	87.51%
Control 5	9.2	28749829	25192799	87.63%
cTKO 1	8.7	35140528	30473193	86.72%
cTKO 2	8.3	31842882	28053570	88.10%
cTKO 3	9.2	37185953	32378392	87.07%
cTKO 4	9.8	28335592	25006294	88.25%
cTKO 5	9.0	34329516	30141227	87.80%

Supplementary Table S1. RNA integrity number (RIN) of RNA samples and mapping rate of reads generated by RNA-seq of the neocortex and hippocampus of controls and cTKO mice at the age of 3 months

	Neocortex			Hippocampus		
Gene name	logFC	p-value	FDR p-value	logFC	p-value	FDR p-value
App	-2.099	7.91E-113	1.10E-108	-1.778	1.26E-80	1.73E-76
Aplp1	-1.921	2.18E-70	1.51E-66	-1.931	1.62E-71	1.11E-67
Aplp2	-1.604	1.76E-54	8.11E-51	-1.247	3.92E-26	7.67E-23
Rskr	-0.836	1.85E-08	1.60E-05	-1.184	1.21E-04	1.14E-02
Npas4	-0.807	5.57E-10	8.56E-07	-1.380	2.13E-08	8.33E-06
Vwa3a	-0.802	2.14E-08	1.74E-05	-1.749	2.79E-13	2.74E-10
Btg2	-0.695	9.35E-08	5.63E-05	-0.484	4.60E-04	2.98E-02
Map3k19	-0.605	7.46E-05	1.13E-02	-0.632	3.36E-04	2.40E-02
Hmgcs2	-0.568	1.23E-04	1.51E-02	-0.714	8.18E-05	8.83E-03
Hrk	-0.520	3.36E-06	1.11E-03	-0.731	5.72E-07	1.51E-04
Nrros	-0.508	2.54E-04	2.59E-02	-0.604	1.81E-04	1.49E-02
Homer2	-0.507	1.85E-05	4.06E-03	-0.585	1.45E-04	1.29E-02
Rims1	-0.493	1.01E-09	1.40E-06	-0.362	7.40E-04	4.04E-02
Ppfia3	-0.478	4.40E-09	4.68E-06	-0.334	4.63E-04	2.98E-02
Cdkl4	-0.451	3.95E-05	7.02E-03	-0.607	1.86E-05	2.66E-03
Hlf	-0.367	4.79E-04	3.95E-02	-0.596	5.42E-04	3.32E-02
Prkcb	-0.332	6.38E-06	1.84E-03	-0.396	7.39E-04	4.04E-02
Tub	-0.292	4.33E-04	3.66E-02	-0.417	4.51E-04	2.98E-02
Ecm1	0.517	3.62E-05	6.68E-03	0.648	5.25E-04	3.28E-02
Npy	0.569	1.11E-04	1.41E-02	1.263	4.22E-07	1.18E-04
Serinc2	0.586	8.25E-05	1.18E-02	1.275	6.98E-09	3.41E-06
Ubtd1	0.630	5.67E-05	9.45E-03	0.581	1.81E-04	1.49E-02
Serpinf1	0.713	5.59E-08	3.69E-05	0.813	8.46E-05	8.99E-03
Blnk	0.718	6.15E-05	9.78E-03	0.824	4.02E-04	2.76E-02
Plekha2	0.724	1.96E-05	4.17E-03	0.840	7.62E-07	1.90E-04
Rab32	0.736	5.16E-04	4.18E-02	0.614	7.85E-04	4.19E-02
Bdnf	0.759	1.44E-05	3.49E-03	1.213	2.48E-08	9.42E-06
Adamts18	0.863	3.44E-04	3.27E-02	1.673	3.96E-16	4.94E-13
Bmp3	0.908	2.42E-08	1.76E-05	1.739	1.99E-11	1.51E-08
Ccnf	0.908	4.28E-04	3.66E-02	1.046	1.64E-05	2.48E-03
Rxfp3	0.945	1.41E-05	3.47E-03	1.064	1.03E-07	3.54E-05
Tll1	1.101	1.06E-07	6.10E-05	0.793	7.60E-04	4.13E-02
Gldn	1.117	2.03E-04	2.14E-02	1.993	3.37E-06	7.00E-04
Als2	1.125	1.04E-40	3.61E-37	2.196	1.62E-35	7.41E-32
Gpnmb	1.161	1.16E-06	4.48E-04	1.727	4.05E-12	3.47E-09
Igsf9	1.199	2.78E-09	3.49E-06	1.007	6.31E-05	7.15E-03
Cxcr4	1.201	5.00E-06	1.54E-03	0.901	8.89E-05	9.37E-03
Col27a1	1.261	7.91E-06	2.18E-03	1.042	5.00E-04	3.14E-02
Prss23	1.306	4.22E-07	1.82E-04	1.072	1.51E-05	2.38E-03
Fndc9	1.372	5.20E-06	1.56E-03	2.342	1.30E-04	1.18E-02
Foxm1	1.505	9.10E-05	1.22E-02	1.299	9.96E-05	1.01E-02
Ecel1	1.532	6.52E-11	1.50E-07	2.161	2.11E-13	2.23E-10
Ly6g6e	2.495	2.29E-08	1.76E-05	1.704	1.56E-07	5.10E-05
Top2a	2.650	6.53E-04	4.81E-02	2.612	1.57E-04	1.37E-02
H19	5.824	6.12E-07	2.57E-04	5.108	8.42E-05	8.99E-03

Supplementary Table S2. List of common DEGs between the neocortex and hippocampus

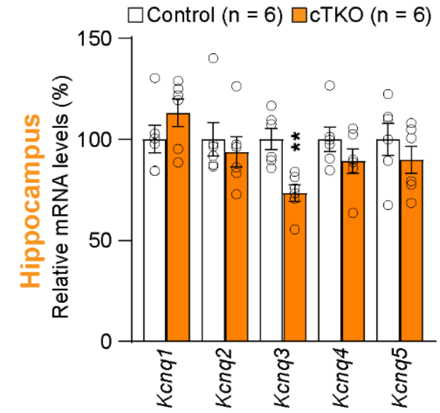

Supplementary Figure S1. Reduced mRNA levels of *Kcnq3* in the hippocampus of cTKO mice

Quantification of the relative expression of the Kv7 channel encoding genes in the hippocampus of cTKO mice and controls at the age of 3 months. Among Kv7 channel encoding genes, only the *Kcnq3* mRNA levels were significantly decreased in cTKO mice compared with controls (26.61%, $p = 0.0024$, Student's *t*-test, $n = 6$ per genotype). The mRNA levels of each gene were normalized to the *Gapdh* and *Rpl13a* housekeeping mRNA levels. All data expressed as means ± SEMs. Statistical analysis was performed with Student's *t*-test. **$p < 0.01$.

Primer ID	Sequence (5'-3')	Target Gene
JKM1698	TTGTCTCCTGCGACTTCA	*Gapdh*-Forward
JKM1699	TCCACCACCCTGTTGCTGTA	*Gapdh*-Reverse
JKM1700	AGGGGCAGGTTCTGGTATTG	*Rpl13a*-Forward
JKM1701	TGTTGATGCCTTCACAGCGT	*Rpl13a*-Reverse
APP-101	GCCATCATCGGACTCATGGT	*APP*-Forward
APP-201	ATCCGTTCTGCTGCATCTTG	*APP*-Reverse
APLP1-101	CTGAGAAGGCCCAGCAGAT	*APLP1*-Forward
APLP1-201	ACCCAAGTGTTCAGCAAGGA	*APLP1*-Reverse
APLP2-101	AGGAACAGCTGGAAATTCGA	*APLP2*-Forward
APLP2-201	GCTGGATGAGGGTCTGTCTT	*APLP2*-Reverse
JKM1482	TGGACATCATTCGCAACTACAC	*Kai1*-Forward-#1
JKM1483	GCATGGGTAAGTGGTCTTGGTA	*Kai1*-Reverse-#1
JKM1484	CTTCAGGAGAACTTCGGCATC	*Kai1*-Forward-#2
JKM1485	AGAATGAATGTACCGGCACAAA	*Kai1*-Reverse-#2
JKM1486	TGGCAGCAAGGTAACCACAG	*Gsk3b*-Forward-#1
JKM1487	CGGTTCTTAAATCGCTTGTCCTG	*Gsk3b*-Reverse-#1
JKM1488	AAGCGATTTAAGAACCGAGAGC	*Gsk3b*-Forward-#2
JKM1489	AGAAATACCGCAGTCGGACTAT	*Gsk3b*-Reverse-#2
JKM1492	GGAACCCATCTCGGCATCC	*Bace1*-Forward-#1
JKM1493	TCCGATTCCTCGTCGGTCTC	*Bace1*-Reverse-#1
JKM1494	CAGTGGGACCACCAACCTTC	*Bace1*-Forward-#2
JKM1495	GCTGCCTTGATGGACTTGAC	*Bace1*-Reverse-#2
JKM1498	TCCCGGTCCAGATCACACTC	*Tip60*-Forward-#1
JKM1499	ACCTTCCGTTTCGTTGAGCG	*Tip60*-Reverse-#1
JKM1500	AGAGAGGTGAAACGGAAGGTG	*Tip60*-Forward-#2
JKM1501	TGGTGCTGACGGTATTCCATC	*Tip60*-Reverse-#2
JKM1576	TCTTGTAAGCAGCCTCAGCC	*Nep*-Forward-#1
JKM1577	ACATAAAGCCTCCCCACAGC	*Nep*-Reverse-#1
JKM1578	ATTCAGCCAAAGCAAGCAGC	*Nep*-Forward-#2
JKM1579	TGAAGAATGGGGGCTGCAAA	*Nep*-Reverse-#2
JKM1510	CCGGGGAGTTGTCTTTCGTG	*P53*-Forward-#1
JKM1511	AATGTGAGGGAAGAGAGTTCCA	*P53*-Reverse-#1
JKM1512	GTCACAGCACATGACGGAGG	*P53*-Forward-#2
JKM1513	TCTTCCAGATGCTCGGGATAC	*P53*-Reverse-#2
JKM1516	GCCATCTGGGCCAAAGATACC	*Egfr*-Forward-#1
JKM1517	GTCTTCGCATGAATAGGCCAAT	*Egfr*-Reverse-#1
JKM1518	GCATCATGGGAGAGAACAACA	*Egfr*-Forward-#2
JKM1519	TCAGGAACCATTACTCCATAGGT	*Egfr*-Reverse-#2
JKM1522	ACTATGGATGCCCCTAAAACTTG	*Lrp1*-Forward-#1
JKM1523	GCAATCTCTTTCACCGTCACA	*Lrp1*-Reverse-#1

JKM1524	CAGAGATGCCCGCCAAATGA	*Lrp1*-Forward-#2
JKM1525	CGTCAAAATCTTTGCACGTCTTG	*Lrp1*-Reverse-#2
JKM1528	AGCCAGTCGGCCATTAACG	*Fe65*-Forward-#1
JKM1529	GCACACTACCCTCTCCCATAG	*Fe65*-Reverse-#1
JKM1530	AGGAGGCCCCAATGGAGTT	*Fe65*-Forward-#2
JKM1531	GCGCACAGCGAAACACTTG	*Fe65*-Reverse-#2
JKM1534	GCTGGAAAATCCCTCGGACA	*vGlut2*-Forward-#1
JKM1535	GCATAGCGGAGCCTTCTTCT	*vGlut2*-Reverse-#1
JKM1536	GTCTTTGGGGCTGCGATACT	*vGlut2*-Forward-#2
JKM1537	CATGACAGGCTGGGTAGGTG	*vGlut2*-Reverse-#2
JKM1584	CTGCTCAACACCCAGGACAT	*X11*-Forward-#1
JKM1585	GTTGGCAGAATGGATCCCCA	*X11*-Reverse-#1
JKM1586	CAGAGAAGTCGGGGAAGCTG	*X11*-Forward-#2
JKM1587	GGTCCGGCCTCCTTATTAGC	*X11*-Reverse-#2
JKM1596	GCACCTCTTCCTTCCCTGAC	*Npas4*-Forward-#1
JKM1597	GATTGGGGCTCAGTTGCTCT	*Npas4*-Reverse-#1
JKM1600	ACCCTGTCACCTTGCAACAA	*Npas4*-Forward-#2
JKM1601	GCAAACCCTTCGTAGGGGAA	*Npas4*-Reverse-#2
JKM1604	TGAGGACACAGAGGCAGGTA	*Rskr*-Forward
JKM1605	GCTGGTCCAGTTCAAAAGCG	*Rskr*-Reverse
JKM999	CACTGACAGAGATCCGCAGAA	*Vwa3a*-Forward
JKM1000	GCAAGCCAGTCTTCAGAGTCT	*Vwa3a*-Reverse
JKM1255	ATGAGCCACGGGAAGAGAAC	*Btg2*-Forward
JKM1256	GCCCTACTGAAAACCTTGAGTC	*Btg2*-Reverse
JKM1211	GGTGGCCTTGGATACTTCTGA	*Map3k19*-Forward
JKM1212	GCACGTCCCTAGATAAGCTACTA	*Map3k19*-Reverse
JKM1203	GAAGAGAGCGATGCAGGAAAC	*Hmgcs2*-Forward
JKM1204	GTCCACATATTGGGCTGGAAA	*Hmgcs2*-Reverse
JKM1608	CGACGAGCTGCACCGA	*Hrk*-Forward
JKM1609	CTACGCGCTCCGCCT	*Hrk*-Reverse
JKM1235	ATTCTGGATGCTAACCCTCTCA	*Nrros*-Forward
JKM1236	GCGTAGTGGCTGATACGGT	*Nrros*-Reverse
JKM1239	CGTTTTGACAGTTTGATACAGCC	*Homer2*-Forward
JKM1240	CAGACACACTCTTTTACGCAGA	*Homer2*-Reverse
JKM561	CAAACCCTAGCCACCCCAG	*Rims1*-Forward
JKM562	CAGGTGTAGATTTGGAGCCAG	*Rims1*-Reverse
JKM1277	GGCCAACTCAAGATGGTGGA	*Ppfia3*-Forward
JKM1278	GGTCTGGCTTTCTTCCCTCC	*Ppfia3*-Reverse
JKM1231	CTGCTGGACAGTGCCTACTT	*Cdkl4*-Forward
JKM1232	CATCAGGTGTGGGGGAGATG	*Cdkl4*-Reverse
JKM983	CCGGCTTCAGCACTATGAAC	*Hlf*-Forward

JKM984	ACGAATGACAATCCTTGTGTCTC	*Hlf*-Reverse
JKM1259	TGGCAAGGTCATGCTCTCAG	*Prkcb*-Forward
JKM1260	TCACAAAGTACAGGCGGTCC	*Prkcb*-Reverse
JKM1263	GGAGCCCCTAGGAAGGAGAA	*Tub*-Forward
JKM1264	AGTCAAGATCTGCACTGGGC	*Tub*-Reverse
JKM1345	TGCCACTACCCTCCTAGTCC	*Ecm1*-Forward
JKM1346	CATGAGGTTGGGGGTGACTC	*Ecm1*-Reverse
JKM194	CTCCGCTCTGCGACACTAC	*Npy*-Forward
JKM195	GGAAGGGTCTTCAAGCCTTGT	*Npy*-Reverse
JKM897	TGCCCACTCCTGGAATCAG	*Serinc2*-Forward
JKM898	ACTCCGTGTAGTAGACGAACAT	*Serinc2*-Reverse
JKM1323	GAGCCCTTGAAGAAGGAGCG	*Ubtd1*-Forward
JKM1324	AGCTCGTCATAGCATTCACAAA	*Ubtd1*-Reverse
JKM1335	GCCCTGGTGCTACTCCTCT	*Serpinf1*-Forward
JKM1336	CGGATCTCAGGCGGTACAG	*Serpinf1*-Reverse
JKM903	GCCCTCCAAGTGTTCCTCG	*Blnk*-Forward
JKM904	GGCAGGCATCACATACATCTC	*Blnk*-Reverse
JKM359	TCTGAAGGACTGGGTAGAAGC	*Plekha2*-Forward
JKM360	CTGATAGGCGTTTGTACCACC	*Plekha2*-Reverse
JKM1610	GCGTGGGTAAGACGAGCATC	*Rab32*-Forward
JKM1611	GTTGAGAACTTTGAGGGCGAA	*Rab32*-Reverse
JKM537	TCATACTTCGGTTGCATGAAGG	*Bdnf*-Forward
JKM538	AGACCTCTCGAACCTGCCC	*Bdnf*-Reverse
JKM1614	TATTTCACACGACATTTTGCACC	*Adamts18-1*-Forward
JKM1615	ACGAGTTCCATTAGAGCAGGG	*Adamts18-1*-Reverse
JKM877	AGCACACGAGCTACCCAAG	*Bmp3*-Forward
JKM878	GTAAGCTGGATACCACGCTTTC	*Bmp3*-Reverse
JKM1375	GAAGGACTTTACAAGCCTGTGT	*Ccnf*-Forward
JKM1376	CCAGGGCGGAAATGATCTCC	*Ccnf*-Reverse
JKM1618	TCCTCATCAGCGCGGTTTAC	*Rxfp3*-Forward
JKM1619	CAGTGCCAGGTTAGTGACAAAG	*Rxfp3*-Reverse
JKM1622	GGTTGGTGGTCTCGGGTATTG	*Tll1*-Forward
JKM1623	GGCGATGTCACCCCAAAACA	*Tll1*-Reverse
JKM1626	ACCAGGCCACAATGGATCAG	*Gldn*-Forward
JKM1627	GGTAGACCCAGTTCACCAGC	*Gldn*-Reverse
JKM032	CCTGGTTATGGGAGGATTCCA	*Als2*-Forward
JKM033	CACACGAAACCTACCACTTCAAA	*Als2*-Reverse
JKM1379	AGCCAATAGGAAACTGCCCC	*Gpnmb*-Forward
JKM1380	TCCTTCTCCTGGTCTCCTCG	*Gpnmb*-Reverse
JKM1295	GGCTGCGCTTTGGATTCCT	*Igsf9*-Forward
JKM1296	GACAACTCTTACCCACGTAATCG	*Igsf9*-Reverse

JKM1630	GAAGTGGGGTCTGGAGACTAT	*Cxcr4*-Forward
JKM1631	TTGCCGACTATGCCAGTCAAG	*Cxcr4*-Reverse
JKM1339	CCTTGGCATCCCATCCAGTT	*Col27a1*-Forward
JKM1340	AAGATCTCCCCTCCCTGGTC	*Col27a1*-Reverse
JKM919	GGTGAGTCCCTACACCGTTC	*Prss23*-Forward
JKM920	GGCGTCGAAGTCTGCCTTAG	*Prss23*-Reverse
JKM1355	AGGAGCCATCATCTCCTGGT	*Fndc9*-Forward
JKM1356	GAAGGAGCGAGGTGTTCCAA	*Fndc9*-Reverse
JKM1636	GCCTGTCTCCTCCACTCCTA	*Foxm1*-Forward
JKM1637	GGGTTCGTACTGGGCTGAAA	*Foxm1*-Reverse
JKM1385	TCTACAACCAGCGCGTGAAT	*Ecel1*-Forward
JKM1386	CACCAGTTCTGTGCAAAGGC	*Ecel1*-Reverse
JKM879	TACTGGTCACGGTCCTACTCT	*Ly6g6e*-Forward
JKM880	GGCAGCATTGCATAGGTCCT	*Ly6g6e*-Reverse
JKM1640	GAGTGCTCGTGTCTCGGAAA	*Top2a*-Forward
JKM1641	TTTGCTTTCTTGCTCGTGGC	*Top2a*-Reverse
JKM1644	CTGCTCCAAGGTGAAGCTGA	*H19*-Forward
JKM1645	TAGAGGCTTGGCTCCAGGAT	*H19*-Reverse
JKM1714	ACCTCATCGTGGTTGTAGCCT	*Kcnq1*-Forward
JKM1715	GGATACCCCTGATAGCTGATGT	*Kcnq1*-Reverse
JKM1460	TACCAGCTTTGGAGGTCTCTT	*Kcnq2*-Forward
JKM1461	ACAGTGTGGAAACACAGCAGA	*Kcnq2*-Reverse
JKM1720	CATCCCAGCAGTCTCCAAGG	*Kcnq3*-Forward
JKM1721	TGCATGTCCACGAGGAAGTC	*Kcnq3*-Reverse
JKM1718	TTGAGCAGTATTCAGCAGGACA	*Kcnq4*-Forward
JKM1719	GGACCCTTATCGCCCTTCTC	*Kcnq4*-Reverse
JKM1470	AAGACTGAGGTTTGCTCGAAAA	*Kcnq5*-Forward
JKM1471	TGTAGGAACCGGAGACTTCTG	*Kcnq5*-Reverse

Supplementary Table S3. List of primer used in this study
